# BroadAMP-GPT: AI-Driven generation of broad-spectrum antimicrobial peptides for combating multidrug-resistant ESKAPE pathogens

**DOI:** 10.1080/19490976.2025.2523811

**Published:** 2025-06-26

**Authors:** Yanru Li, Xianghan Xu, Xiaohui Zhang, Zhihui Xu, Jiaqi Zhao, Ruiyu Zhu, Ziyu Wang, Wei Ran, Wenqian Zhao, Ningyang Yan, Yifan Leng, Zexu Miao, Xiaomin Wang, Liping Wang, Jinxin Liu, Cong Pian, Jinhu Huang

**Affiliations:** a Sanya Institute of Nanjing Agricultural University, Nanjing Agricultural University, Sanya, China; b College of Agriculture, Nanjing Agricultural University, Nanjing, Jiangsu, China; c MOE Joint International Research Laboratory of Animal Health and Food Safety, Risk Assessment Center of Veterinary Drug Residue and Antimicrobial Resistance, Center for Veterinary Drug Research and Evaluation, College of Veterinary Medicine, Nanjing Agricultural University, Nanjing, China; d Department of Veterinary Medicine, School of Tropical Agriculture and Forestry, Hainan University, Haikou, China; e School of Basic Medicine and Clinical Pharmacy, China Pharmaceutical University, Nanjing, Jiangsu, China; f Laboratory of Gastrointestinal Microbiology, Jiangsu Key Laboratory of Gastrointestinal Nutrition and Animal Health, National Center for International Research on Animal Gut Nutrition, College of Animal Science and Technology, Nanjing Agricultural University, Nanjing, Jiangsu, China; g College of Sciences, Nanjing Agricultural University, Nanjing, Jiangsu, China

**Keywords:** Antimicrobial peptides, AMP generation and screening, broad-spectrum activity, ESKAPE pathogens, experimental validation

## Abstract

Antimicrobial peptides (AMPs) are promising candidates to address the global antimicrobial resistance crisis, yet their traditional design remains labor-intensive and inefficient. Here, we developed BroadAMP-GPT, an integrated computational-experimental framework that combines AI-driven generation, multi-tiered screening, and experimental validation to rapidly discover potent AMPs with broad-spectrum activity. Using this platform, 57% of AI-generated candidates exhibited potent efficacy against ESKAPE pathogens – key culprits of multidrug-resistant infections. An outstanding candidate, AMP_S13, demonstrated exceptional stability under diverse physiological conditions, including extreme pH (2–10), proteolytic exposure, and elevated temperatures, while maintaining minimal cytotoxicity and low hemolytic activity. AMP_S13 also showed robust *in vivo* efficacy, reducing mortality in *Galleria mellonella* infection model and accelerating wound healing in a murine MRSA skin infection model. These results validate BroadAMP-GPT as a transformative tool for accelerating the discovery of stable, broad-spectrum and low-toxicity AMPs, offering a scalable strategy to address the urgent threat of multidrug-resistant pathogens.

## Introduction

The global health crisis posed by antimicrobial resistance (AMR) continues to escalate,^1^ with multidrug-resistant (MDR) bacteria – notably the ESKAPE pathogens (*Enterococcus faecium*, *Staphylococcus aureus*, *Klebsiella pneumoniae*, *Acinetobacter baumannii*, *Pseudomonas aeruginosa*, and *Enterobacter* spp.) – directly responsible for 1.27 million deaths annually and contributing to 4.95 million fatalities worldwide.^2^ Current projections estimate AMR-related mortality to reach 10 million deaths per year by 2050,^3^ demanding urgent development of therapies that circumvent existing resistance mechanisms.

Antimicrobial peptides (AMPs), with their ability to disrupt microbial membranes through pore formation and lytic activity, represent a promising alternative to conventional antibiotics.^4^ These amphiphilic peptides, typically 10–50 amino acids (AAs) in length, exhibit broad-spectrum activity against MDR pathogens, while minimizing resistance development due to their physicochemical complexity.^5^ However, the vast sequence space of AMPs (~20^15 possible 20-mer combinations) and labor-intensive screening pipelines hinder their rapid discovery.

Artificial intelligence (AI) has emerged as a transformative tool for AMP design, yet current approaches face critical limitations.^6,7^ Current generative models, such as Generative Adversarial Networks (GANs)^8,9^ and Variational Autoencoders (VAEs),^10–12^ struggle to capture long-range sequence dependencies and prone to training instability and pattern collapse, resulting in peptides with low diversity and compromised stability under physiological conditions.^13,14^ Similarly, conventional screening pipelines – whether machine learning (ML)-based methods (e.g., Support Vector Machines (SVM)^15,16^ and Random Forest (RF)^15–17^) or deep learning (DL)-based approaches (e.g., Convolutional Neural Networks (CNNs), Long Short-Term Memory (LSTM) networks, and Graph Attention Networks (GATs)^18,19^) – often rely on oversimplified descriptors (e.g., AA composition, physicochemical properties) that fail to model complex sequence-activity relationships.^20^ Moreover, few platforms integrated AI-driven generation with experimental validation of therapeutic potential.

To address these challenges, we developed BroadAMP-GPT, an AI-powered framework that synergizes transformer-based generation, deep learning-guided screening, and multi-modal experimental validation to accelerate the discovery of stable, broad-spectrum AMPs. From AI-generated candidates, 57% demonstrated potent activity against ESKAPE pathogens. A lead peptide, AMP_S13, exhibited broad-spectrum efficacy, robust stability across extreme conditions, minimal cytotoxicity, and low hemolytic activity. *In vivo*, AMP_S13 reduced mortality in *Galleria mellonella* infection model and accelerated MRSA-infected wound healing in mice. By bridging AI innovation with rigorous experimental validation, BroadAMP-GPT establishes a scalable paradigm for combating the global AMR crisis.

## Results

### BroadAMP-GPT: an integrated computational and experimental framework for designing high-potency, broad-spectrum AMPs

**Figure 1.**
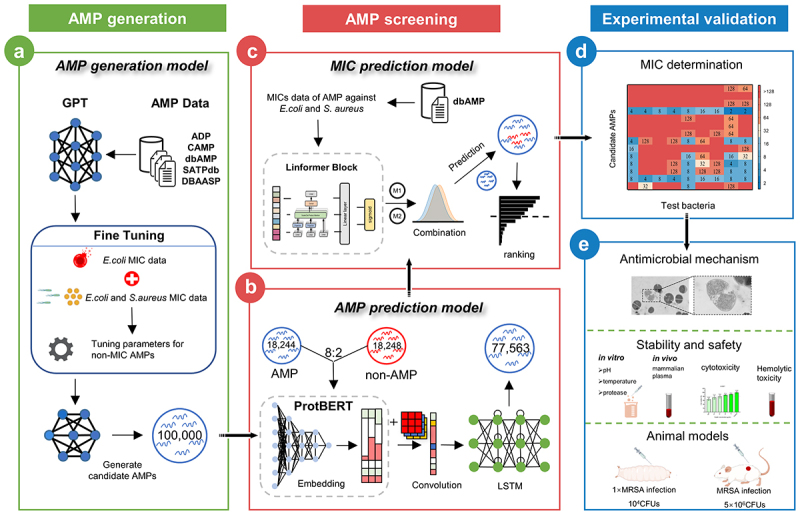
BroadAMP-GPT overview. The framework consists of three modules: (a) AMP generation module consisting of AMP generation model. AMP sequences from the five databases were collected and used to train AMP generation model; (b-c) AMP screening module consisting of the AMP prediction model and two MIC prediction models. AMP dataset and MIC dataset were constructed and used to train binary classification models, which was then applied to screen generated peptide sequences. The screening was completed by combining AMP prediction model (B), *E. coli* MIC prediction model and *S. aureus* MIC prediction model (c); (d-e) experimental validation module consisting chemical synthesis of candidate AMPs, MIC testing antimicrobial mechanism, stability, safety and animal models were performed in the wet laboratory. The MIC value unit is μg/mL.

A novel framework, designated BroadAMP-GPT, has been developed in the present study for the *de novo* design of AMPs with high potent and broad-spectrum activity against MDR pathogens. This framework integrates three distinct modules: AMP generation, screening, and experimental validation (Figure 1).

To establish a robust AMP generation pipeline, we compiled AMP sequences from five publicly available databases (dbAMP,^21^ APD,^22^ CAMP,^23^ SATPdb^24^ and DBAASP^25^) into a comprehensive dataset comprising 26,545 AMP sequences (pre-trained AMP dataset) for pre-training and 3,682 AMP sequences with contain *Escherichia coli* and *Staphylococcus aureus* MIC data (fine-tuning dataset) for fine-tuning (See Methods). A GPT-based^26^ model was pre-trained using the pre-trained AMP dataset and subsequently fine-tuned with the fine-tuning dataset to tailor the model for generating peptides with broad-spectrum antimicrobial properties, specifically targeting *E. coli* and *S. aureus* (Figure 1(a)). Using this trained model, we generated 100,000 peptide sequences for further analysis.

To identify candidate AMPs with high potency and broad-spectrum activity, we implemented a two-step screening process using DL-based prediction models (Figure 1(b,c)). First, an AMP prediction model was developed and trained on AMP and non-AMP datasets (See Methods). Input sequences were encoded using the protein pre-trained model ProtBERT^27^ followed by feature extraction via a convolutional (Conv) layer and LSTM layer. The extracted features were fed into a fully connected (FC) layer, which output probability values. A stringent probability threshold of 0.998 was applied, resulting in the selection of 77,563 high-potency peptide sequences (Figure 1(b)). Next, a low-rank self-attention model, Linformer,^28^ was employed to construct MIC prediction models. These models were trained separately using *E. coli* and *S. aureus* MIC datasets, producing MIC prediction models for both Gram-negative and Gram-positive bacteria, respectively (Figure 1(c)). Screening these high-potency sequences (length <25 AAs) with the MIC prediction models yielded 110 candidate AMPs with broad-spectrum activity targeting MDR bacteria, including the ESKAPE pathogens.

Fourteen candidate AMPs were selected from the computationally screened peptide pool for experimental validation. Antimicrobial activity was evaluated by determining the MICs of these candidates against *S. aureus* ATCC 29,213, *E. coli* ATCC 25,922, and other MDR bacteria, including ESKAPE pathogens (Figure 1(d)). Subsequent analyses included investigations into the antimicrobial mechanisms, stability under various conditions, cytotoxicity, and *in vivo* efficacy (Figure 1(e)). Experimental validation demonstrated that BroadAMP-GPT effectively generates AMPs with high-potency and broad-spectrum activity against ESKAPE pathogens.

### Screening the generated peptide sequences to identify candidate AMPs

To identify high-potency and broad-spectrum AMPs from the generated peptide sequences, we developed and implemented several predictive models, including an AMP prediction model, an *S. aureus* MIC prediction model, and an *E. coli* MIC prediction model.

**Figure 2.**
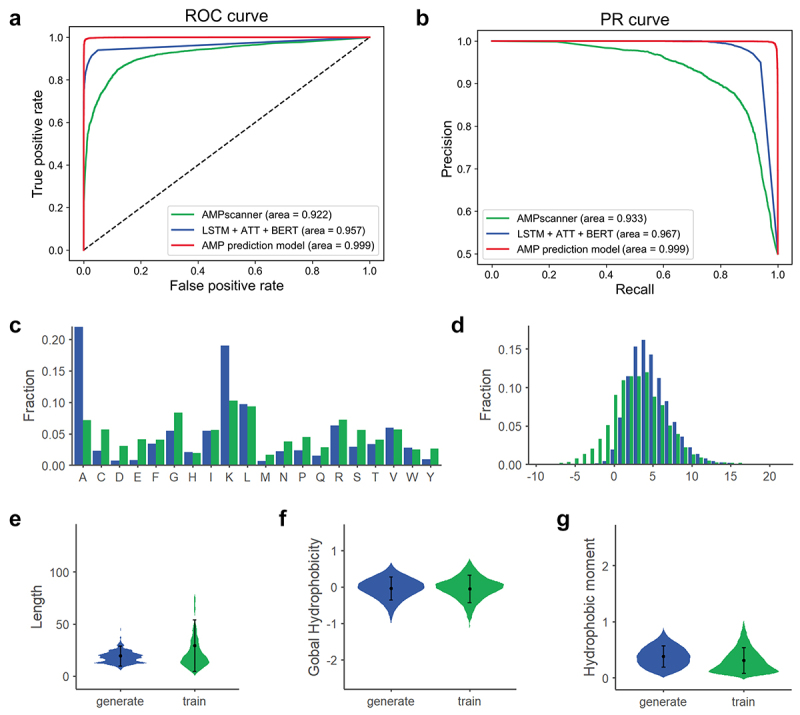
AMP prediction model performance and physicochemical properties of the generated peptide sequences. (a) AUROCs for different AMP prediction models. (b) AUPRCs for different AMP prediction models. (c–g) the distribution of aas, charge, length, global hydrophobicity and hydrophobic moment for generate and train sequences attributes.

We evaluated the performance of our AMP prediction model against two existing methods: the natural language processing (NLP)-based LSTM+ATT+BERT^19^ framework and the DL-driven AMPscanner.^18^ As shown in Figure 2(a,b) and Supplementary Table S1, our model achieved superior performance on the test dataset compared to these benchmark approaches. Specifically, our model achieved higher area under the receiver operating characteristic curve (AUROC; improvement of 4.2–7.7%) and area under the precision-recall curve (AUPRC; improvement of 3.2–6.6%). Additionally, the AMP prediction model exhibited superior performance compared to AMPscanner across all metrics, including accuracy (ACC), sensitivity (Sens), specificity (Spec), Matthews correlation coefficient (MCC), and F1 score. Compared to LSTM+ATT+BERT, our model achieved higher sensitivity (31% improvement), MCC (27% improvement), F1 score (18% improvement) and, ACC (15% improvement), with only slightly lower in specificity. These results confirm the robustness of our AMP prediction model for identifying AMPs from sequence data.

To further evaluate the quality of the sequences screened by the AMP prediction model, we compared their physicochemical properties with those of the training dataset. Key molecular features, such as AA composition, charge, length, hydrophobicity, and hydrophobic moment, were analyzed (Figure 2(c–g)). As shown in Figure 2(c), statistical analysis revealed that the generated peptides exhibit physicochemical distributions comparable to those in the training dataset, indicating that our AMP generation model successfully captured the fundamental features of natural AMPs. Importantly, while preserving the characteristics of natural AMPs, the model enhances key antibacterial activity features including positive charge (enriched positively charged AAs: Lys (K)) and amphipathic structures (enriched hydrophobic AAs: Ala (A)). Figure 2(d) further demonstrates that the generated peptide sequences display significantly higher average charge compared to the training sequences, which represents a critical feature for most AMPs. In terms of length, the generated peptide sequences were shorter (Figure 2(e)), making them more suitable for synthesis. The hydrophobicity and hydrophobic moment of the generated and training sequences were comparable (Figure 2(f,g)) because the sequences were generated through the composition distribution of training. Collectively, these findings indicate that the generated sequences possess the essential physicochemical properties of cationic charge and amphiphilicity while exhibiting low sequence similarity with the training dataset, highlighting their novelty.

For the MIC prediction models, using the same modeling framework as for AMP model would lead to overfitting due to the small amount of training data. We adopted the Linformer framework,^28^ an efficient Transformer based on the idea of low-rank self-attention and reduces the overall self-attention complexity. This framework was used to train separate MIC prediction models for *E. coli* and *S. aureus*. The *E. coli* MIC prediction model achieved a mean AUROC of 0.77 on the validation set, while the *S. aureus* MIC prediction model achieved a mean AUROC of 0.71 (Supplementary Figure S1). The best-performing parameters from cross-validation were used to finalize the prediction models.

After screening the generated peptide sequences with the MIC prediction models, 110 candidate AMPs with high potency and broad-spectrum activity were identified. We randomly selected a subset of 15 peptides for experimental validation. Due to one failed synthesis quality control, 14 peptides were ultimately used for validation. To evaluate the homology between the candidate AMPs and existing database entries, we performed BLAST sequence similarity searches. The expect value (E-value) served as primary metric for evaluating sequence homology, indicating the statistical significance of matches between query sequences and a database. A large E-value suggests non-homology between the hit and the query.^29^ E-value thresholds were set according to the database scale: 0.001 for UniProt^30^ and 1 × 10^− 6^ for the training dataset.^31^ Supplementary Tables S2 and S3 demonstrate that in the alignment results between candidate AMPs and UniProt, all E-values were greater than 0.001, while in the alignments between candidate AMPs and the training dataset, all E-values exceeded 1 × 10^−6^, indicating no detectable homology to any known sequences. The results demonstrate that the candidate AMPs exhibit unique characteristics.

### Candidate AMPs demonstrate high-potency and broad-spectrum activity against ESKAPE pathogens and other MDR bacteria

**Figure 3.**
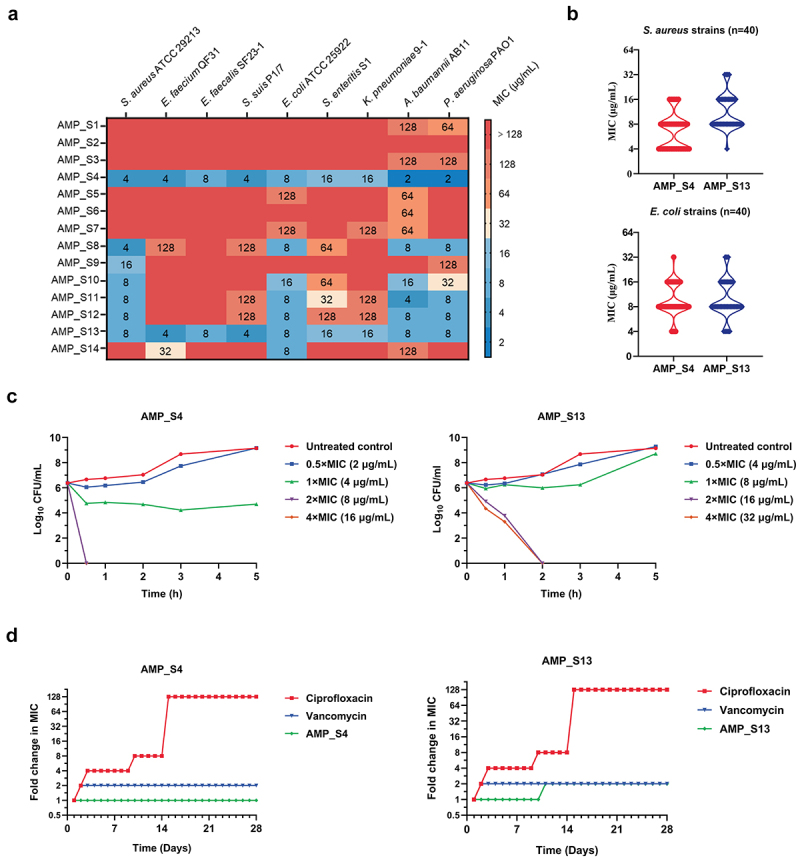
Candidate AMPs antimicrobial activity and resistance-acquisition experiments. (a) MICs of AMP_S4 and AMP_S13 against diverse bacterial species. (b) MIC_s_ of AMP_S4 and AMP_S13 against *S. aureus* (*n* = 40) and *E. coli* (*n* = 40). (c) Time-killing curves of AMP_S4 and AMP_S13 against *S. aureus* ATCC 29,213. (d) Resistance-acquisition studies of *S. aureus* ATCC 29,213 in the presence of sub-MIC (1/2×) levels of ciprofloxacin, vancomycin, AMP_S4 and AMP_S13.

To assess the antimicrobial activity of the generated AMPs, 14 candidates were synthesized and tested against representative Gram-positive (*S. aureus* ATCC 29,213) and Gram-negative (*E. coli* ATCC 25,922) bacteria. Six candidates (AMP_S4, S8, S10, S11, S12, S13) exhibited strong activity against both strains, while AMP_S9 and AMP_S14 were either active against *S. aureus* or *E. coli* (Figure 3(a)). Testing on 40 clinical isolates each of *S. aureus* and *E. coli* revealed MICs ranging from 4 to 32 μg/mL for AMP_S4 and AMP_S13, confirming their consistent efficacy and validating the MIC model predictions (Figure 3(b)).

Due to the shared cell wall architecture of Gram-positive and Gram-negative bacteria, AMP_S4 (AKKWWKLLWKLWKKAK) and AMP_S13 (AWQAIARLIKGRGRKKYGRIIIIG) were further tested against a broader panel of pathogens. Both AMPs demonstrated potent activity (MICs: 2–16 μg/mL) against four Gram-positive and five Gram-negative bacteria, including ESKAPE pathogens (Figure 3(a)). Additional testing against antibiotic-susceptible and -resistant strains confirmed their broad-spectrum efficacy, as summarized in Table 1. These results emphasize the effectiveness of AMP_S4 and AMP_S13 against MDR bacteria and underscore the utility of the BroadAMP-GPT framework for generating therapeutically relevant AMPs.

**Table 1.** Mics of candidate AMPs against ESKAPE pathogens and other specific antibiotic-susceptible and antibiotic-resistant strains.

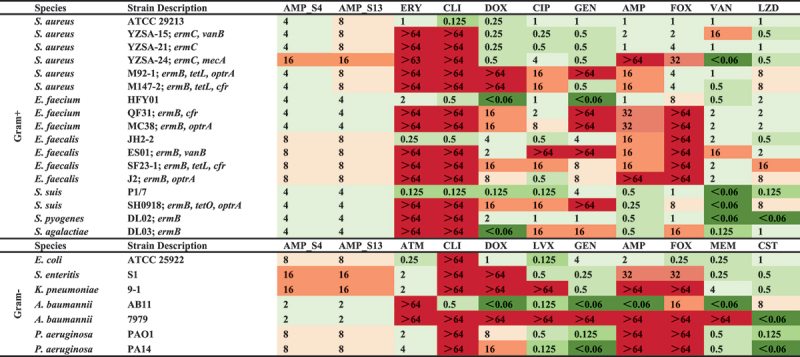

AMPs in comparison with clinically approved antibiotics. Abbreviations: ERY, Erythromycin; CLI, clindamycin; DOX, doxycycline; CIP, ciprofloxacin; GEN, gentamicin; AMP, Ampicillin; FOX, Cefoxitin; VAN, vancomycin; LZD, linezolid; ATM, Aztreonam; LVX, Levofloxacin; MEM, Meropenem; CST, colistin *vanB*, vanB-type vancomycin-resistance locus; *ermB/C*, erythromycin-resistance rRNA methylase B/C; *mecA*, methicillin-resistance gene; *cfr*, chloramphenicol-florfenicol resistance methylase; *optrA*, oxazolidinone phenicol transferable resistance gene; *tetL/O*, tetracycline efflux protein L/O. unit: μg/mL.

The bactericidal properties of AMP_S4 and AMP_S13 were further characterized by determining their MBCs, yielding MBC/MIC ratios ≤4 μg/mL, consistent with strong bactericidal activity (Supplementary Table S4). No significant differences were observed in bactericidal effects between antibiotic-susceptible and -resistant strains. Time-kill assays with *S. aureus* ATCC 29,213 revealed dose-dependent killing effects for both AMPs. At 1×MIC, significant bacterial reductions were observed within 3 hours, while 2 × MIC achieved complete bacterial eradication within 30 minutes and 2 hours for AMP_S4 and AMP_S13, respectively (Figure 3(c)).

Resistance-acquisition experiments with *S. aureus* ATCC 29,213 exposed to sub-MIC concentrations of AMP_S4 and AMP_S13 demonstrated no detectable resistance after 28 passages. In contrast, ciprofloxacin induced resistance after only nine passages (Figure 3(d)). These findings highlight the low propensity of AMP_S4 and AMP_S13 to induce resistance, a critical advantage for therapeutic applications targeting MDR bacteria. In summary, AMP_S4 and AMP_S13 exhibit potent, broad-spectrum activity against ESKAPE pathogens and other MDR bacteria, with rapid bactericidal effects and minimal resistance development. These features underscore their potential as promising therapeutic agents warranting further investigation.

### AMP_S4 and AMP_S13 specifically target bacterial cell membrane

**Figure 4.**
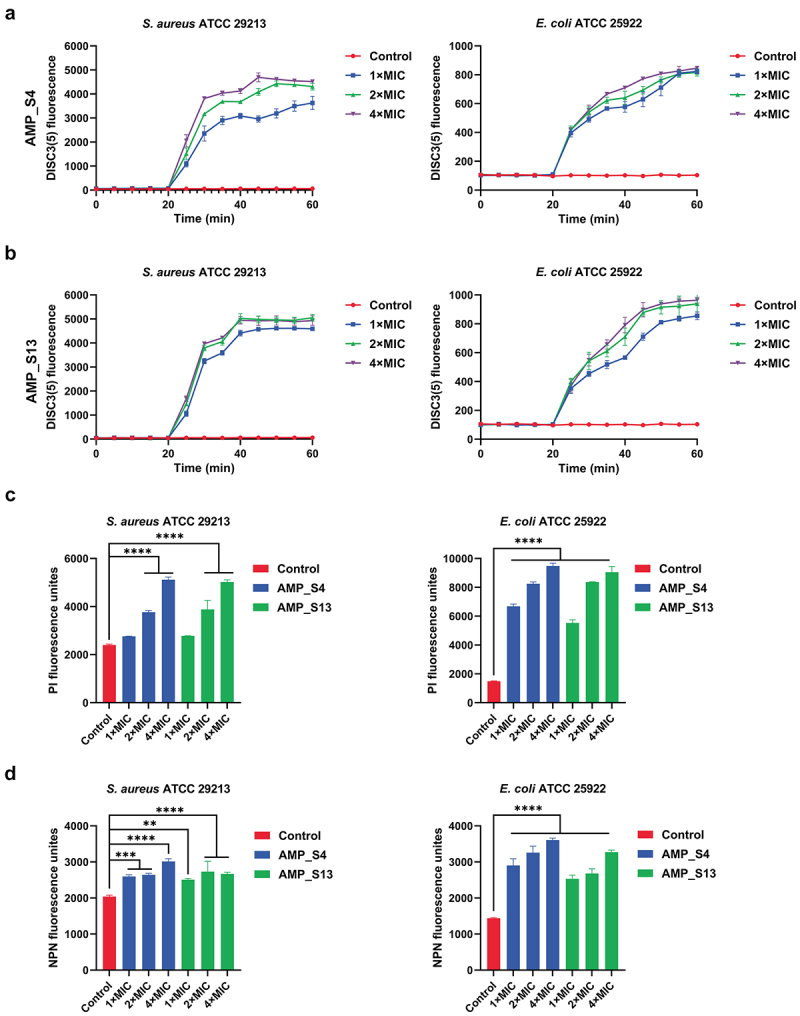
Amps-induced membrane-destabilizing effects. Cytoplasmic membrane depolarization induced by AMP_S4 (A) and AMP_S13 (b) against *S. aureus* ATCC 29,213 and *E. coli* ATCC 25,922 at 1×MIC, 2×MIC, and 4×MIC concentrations. (c) Outer membrane permeability induced by AMP_S4 and AMP_S13 (1×MIC, 2×MIC, and 4×MIC) was detected by NPN uptake in *S. aureus* ATCC 29,213 and *E. coli* ATCC 25,922. The enhanced uptake of NPN was measured by the increased fluorescence of NPN. (d) the inner membrane rupture induced by AMP_S4 and AMP_S13 was detected by the increased fluorescence of PI in *S. aureus* ATCC 29,213 and *E. coli* ATCC 25,922 at concentrations of 1×MIC, 2×MIC, and 4×MIC. Data were reported as the mean ± SD (*n* = 3 per group) from three independent trials; significant differences compared to the control group were calculated by one-way ANOVA and Dunnett’s test (ns, not significant; *p < 0.5; **p < 0.01; ***p < 0.001; ****p < 0.0001).

The determination of membrane depolarization and permeability provides a basis for exploring the antibacterial mechanisms of AMP_S4 and AMP_S13. Using the DiSC3(5) probe, both peptides induced dose-dependent increases in fluorescence intensity in *S. aureus* ATCC 29,213 and *E. coli* ATCC 25,922, indicating disruption of the proton motive force (PMF) (Figure 4(a,b)). Outer membrane permeability assays with the NPN probe revealed fluorescence intensity enhancements, consistent with outer membrane disruption (Figure 4(c)). Inner membrane integrity assays with propidium iodide (PI) showed increased fluorescence intensity after one hour of peptide treatment, proportional to peptide concentration (Figure 4(d)).

**Figure 5.**
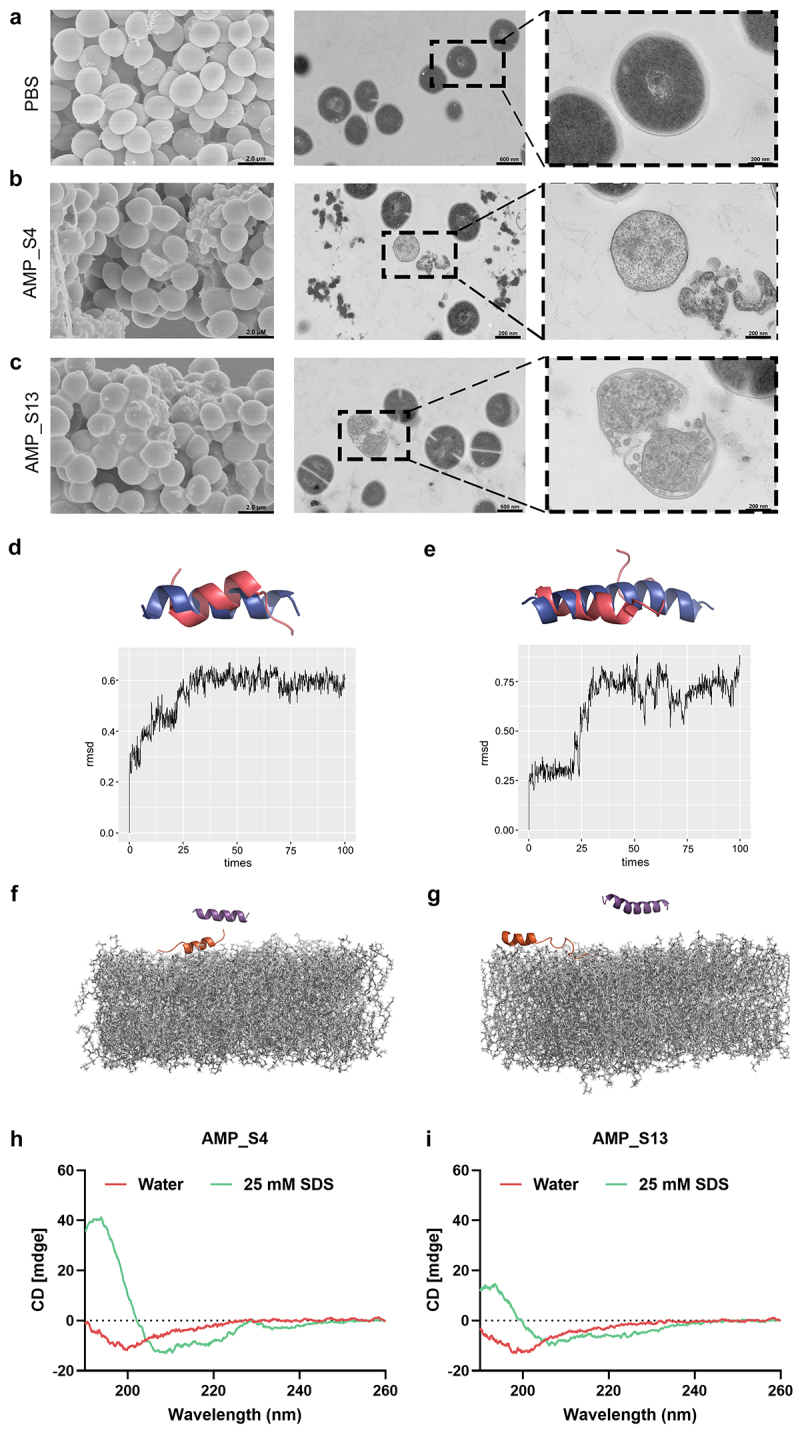
(a–c) Electron microscopy images of bacteria treated with PBS. (a), AMP_S4 (b) and AMP_S13 (c). The middle column is TEM images at low magnification (scale bar, 600 nm). The right column is TEM images at high magnification (scale bar, 200 nm). The left column is SEM images (scare bar 2.0 µM). (d-g), MD simulation of AMP_S4 and AMP_S13. (d-e) the structures predicted by AlphaFold2 (in blue) was aligned with the structure from MD simulations (in red) performed at 100 ns in water box. D, AMP_S4, E, AMP_S13. (f-g) the structures from MD simulations of peptide–membrane interactions. F, AMP_S4 performed with membrane at 0 ns (in purple). AMP_S4 performed with membrane at 85 ns (in orange). G, AMP_S13 performed with membrane at 0 ns (in purple). AMP_S13 performed with membrane at 60 ns (in orange). (h-i) Structural analyses of the AMP_S4 and AMP_S13 in water and 25 mm SDS. The final spectra were the average of 3 scans after subtracting the spectrum obtained under the same conditions of a sample without peptide.

To investigate whether AMP_S4 and AMP_S13 exert their antimicrobial effects by targeting bacterial membranes, transmission electron microscopy (TEM) and scanning electron microscopy (SEM) were employed. Control *S. aureus* ATCC 29,213 cells displayed intact, smooth membranes (Figure 5(a)). However, treatment with AMP_S4 and AMP_S13 at 2×MIC for 2 hours caused significant membrane disruption and lysis, as evidenced by TEM and SEM (Figure 5(a–c)). These results confirm that AMP_S4 and AMP_S13 exert their bactericidal effects through pronounced membrane deformation and disruption.

To further validate the membrane-targeting mechanism, molecular dynamics (MD) simulations were conducted in aqueous solution and in the presence of bacterial membranes. After 100 ns of simulation in aqueous solution, both AMPs retained predominantly α-helical structures, with minor unstructured regions at their termini (Figure 5(d,e)), suggesting structural stability contributes to their antimicrobial efficacy. In the presence of bacterial membranes, MD simulations revealed distinct membrane-binding mechanisms. AMP_S4 utilized positively charged N-terminal residues (Ala, Lys) for membrane embedding, while AMP_S13 relied on C-terminal Gly residues (Figure 5(f,g)). These interactions, driven by polar residues, offer insights into their distinct modes of action and the low likelihood of resistance development due to the nonspecific nature of membrane targeting.

Subsequently, we conducted experimental assessments of the secondary structure of the active AMPs using circular dichroism (CD). In 25 mm SDS, the CD spectrum of both AMPs showed a positive peak at approximately 195 nm and two negative peaks near 208 and 222 nm (Figure 5(h,i)), which are characteristic profiles of α-helical structures. The α-helical content was significantly higher in SDS micelles than in water, increasing from 17.1% to 62.4% for AMP_S4 and from 16.7% to 29.2% for AMP_S13 indicating that they undergo helical transition upon contact with hydrophobic/hydrophilic interfaces, such as bacterial membranes.

These findings collectively demonstrate that AMP_S4 and AMP_S13 disrupt bacterial membranes by destabilizing both outer and inner membrane structures. This membrane-targeting mechanism underpins their rapid bactericidal effects and highlights their potential as novel antimicrobial agents.

### AMP_S13 exhibits excellent stability and safety profiles

**Figure 6.**
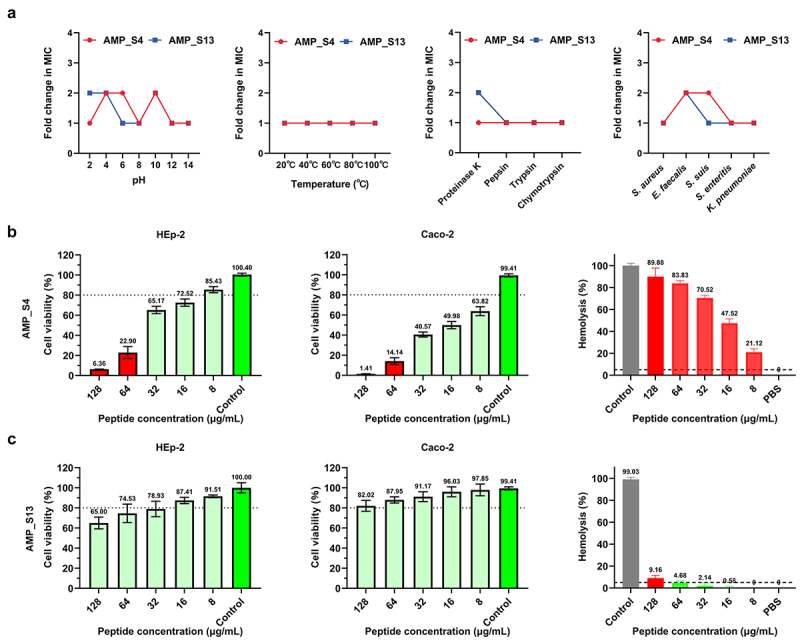
Stability and toxicity of AMP_S4 and AMP_S13. (a) Analysis of the effect of pH, temperature, proteases and plasma on AMP_S4 and AMP_S13 antimicrobial activity. The peptides were incubated at different conditions for 1 h followed by examine the MICs. Cytotoxicity of HEp-2, Caco-2 and haemolytic toxicity of mice red blood cell of AMP_S4 (b) and AMP_S13 (c). (Values were presented as the mean ± SD, *n*=3).

The stability of AMP_S4 and AMP_S13 was assessed under various *in vitro* (pH, temperature, protease) and *in vivo* (mammalian plasma) conditions by comparing MIC and MBC fold changes. Both AMPs exhibited stable antimicrobial activity against *S. aureus* ATCC 29,213 across a pH range of 2 to 14, with less than a two-fold change in MICs (Figure 6(a)). They also retained activity at temperatures ranging from 20°C to 100°C. Protease stability tests revealed that AMP_S13 maintained its activity after incubation with proteinase K, pepsin, trypsin, and chymotrypsin with only a two-fold decrease in MIC when exposed to proteinase K (Figure 6(a)). Additionally, in the presence of 50% rabbit plasma, AMP_S13 remained effective against various bacterial strains, including *S. aureus*, *E. faecalis*, *S. suis*, *S. enteritis*, and *K. pneumoniae* (Figure 6(a)), demonstrating robust plasma stability.

The cytotoxicity of AMP_S4 and AMP_S13 was evaluated using HEp-2 and Caco-2 mammalian cell lines via the CCK-8 assay. AMP_S4 exhibited higher toxicity, with CC50 values of 35.16 µg/mL and 15.82 µg/mL for HEp-2 and Caco-2, respectively (Figure 6(b,c) Supplementary Table S5). In contrast, AMP_S13 demonstrated significantly lower toxicity, with CC50 values of 353.3 µg/mL and 1011 µg/mL, respectively, highlighting its superior biocompatibility. The selective index (SI = hC50/MIC) further confirmed the favorable safety profile of AMP_S13 compared to AMP_S4.

Hemolytic toxicity tests on mouse red blood cells showed that AMP_S4 caused 21.12% hemolysis at a concentration of 8 µg/mL, with an HC50 of 18.25 µg/mL (Figure 6(d), Supplementary Table S5). In contrast, AMP_S13 displayed negligible hemolytic activity at the same concentration, with an HC50 of 928.6 µg/mL. These findings indicate that AMP_S13 has minimal hemolytic toxicity and selective toxicity, making it a safer candidate for therapeutic applications.

### AMP_S13 displays robust in vivo efficacy in MRSA-infected animal models

**Figure 7.**
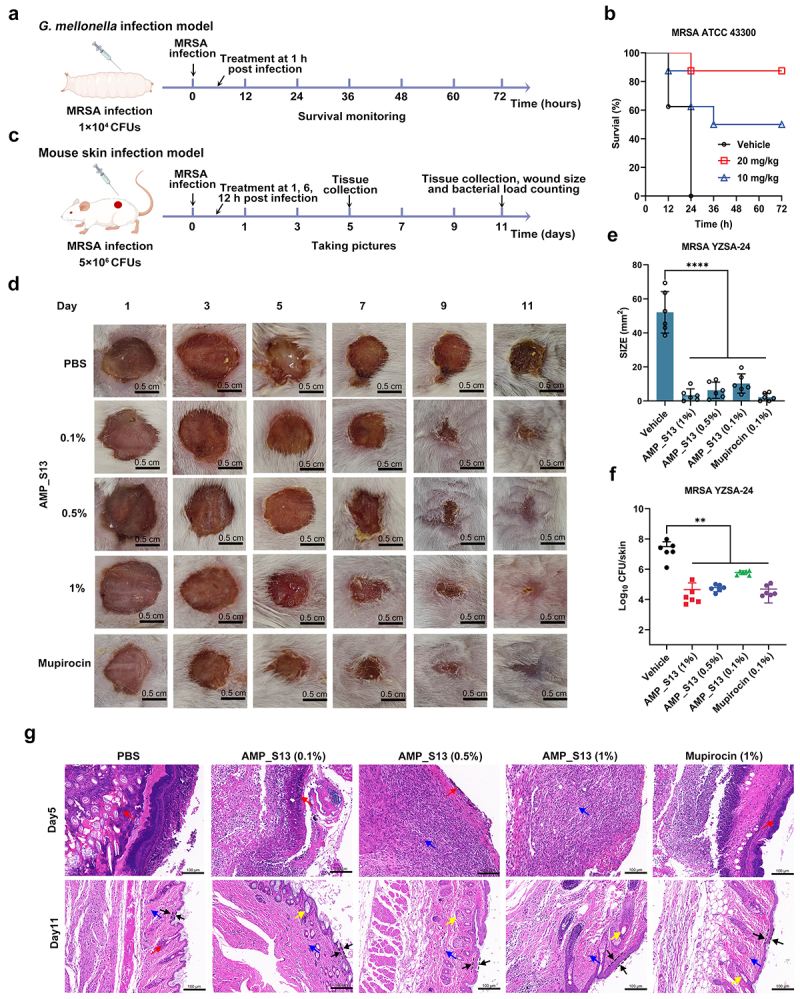
*In vivo* antimicrobial efficacy of AMP_S13 in *Galleria mellonella*larvaeinfection model and skin wound infection models. (a) Schematic depiction of MRSA-infected *Galleria mellonella* model employed in the animal trial. (b) Percent survival of MRSA-infected *Galleria mellonella* treated with AMP over the 72 h of the study. (c) Schematic depiction of mouse skin infection model employed in the animal trial (d)Typical photographs of the skin wound at day 1, 3 ,5, 7, 9, and 11 after different treatment. (e) Size of the skin wounds and (F) bacterial load of the skin wounds after 11 days. All the data are presented as the means±SDs, and the significance of the differences was determined via one-way ANOVA (*p < 0.05, **p < 0.01, ***p < 0.001, ****p < 0.0001). (g) Histologic analysis of MRSA-infected skin tissue by hematoxylin and eosin (H&E) staining for different treating groups. The red arrows point to fibroblasts, the blue arrows point to fibroblasts, the yellow arrows point to hair follicles, the black arrows point to epidermis.

Building on its favorable *in vitro* profile, the *in vivo* antimicrobial efficacy of AMP_S13 was evaluated using *Galleria mellonella* larvae and mouse skin infection models (Figure 7). In the *Galleria mellonella* model infected with MRSA ATCC 43,300 (Figure 7(a)), AMP_S13 treatment resulted in a dose-dependent increase in survival. At a dosage of 10 mg/kg, 50% of larvae survived, while increasing the dose to 20 mg/kg improved survival rates to 90% (Figure 7(b)), demonstrating strong protective effects against lethal MRSA infections.

In the mouse skin infection model infected with the MRSA clinical isolate YZSA-24 (Figure 7(c)), AMP_S13 treatment significantly reduced wound suppuration and accelerated wound healing, particularly at higher doses (Figure 7(d)). Quantitative analysis after 11 days of treatment revealed that AMP_S13 markedly reduced wound size and bacterial load compared to untreated controls (Figure 7(e,f)). Notably, the therapeutic effect of 1% AMP_S13 was comparable to mupirocin, a widely used topical antibiotic for treating Gram-positive bacterial infections.

Histological analysis of infected skin tissues (Figure 7(g)) further supported the efficacy of AMP_S13. On day 5, PBS-treated control mice showed pronounced neutrophil infiltration, extensive skin surface loss, and hair follicle destruction. In contrast, mice treated with AMP_S13 exhibited milder inflammatory responses. By day 11, PBS-treated mice displayed significant epithelial defects and a lack of hair follicle regeneration, whereas AMP_S13-treated mice showed enhanced re-epithelialization and significantly increased hair follicle and sebaceous gland formation in a dose-dependent manner. These findings underscore the strong *in vivo* antimicrobial efficacy of AMP_S13, highlighting its potential as a therapeutic agent for MRSA infections.

## Discussion

In this study, we developed an AI-guided framework, BroadAMP-GPT, that integrates the GPT model^26^ with DL architectures to facilitate the *de novo* design and screening of high-potency, broad-spectrum AMPs targeting ESKAPE pathogens. The experimental validation revealed that AMP_S13 exhibited exceptional broad-spectrum antibacterial activity, remarkable stability under diverse conditions, minimal cytotoxicity to mammalian cells, and low hemolytic activity. Additionally, AMP_S13 demonstrated robust *in vivo* antimicrobial efficacy in animal models, underscoring its therapeutic potential.

While AI-based approaches for AMP design have been proposed,^12,29,32,33^ BroadAMP-GPT stands out as a comprehensive and fully automated platform for broad-spectrum AMP discovery. Unlike traditional methods, it supports controlled generation and screening and can handle multiple controls simultaneously. BroadAMP-GPT has additional advantages regarding repurposability, as adding a new condition requires a simple predictor training. The use of GPT for AMP generation mitigates issues associated with the instability of GAN training and the spatial distribution constraints of VAE models. Furthermore, the inclusion of two innovative MIC prediction models in the screening module allowed us to identify AMPs with broad-spectrum antimicrobial activity. Due to the limited availability of experimental MIC data for certain ESKAPE pathogens, we focused on *E. coli* (Gram-negative) and *S. aureus* (Gram-positive) as representative organisms for MIC prediction. Overlapping sequences identified by both models were deemed to exhibit broad-spectrum antimicrobial activity.

Among the 14 synthesized candidate AMPs, six demonstrated strong activity (MIC ≤16 μg/mL) against both *E. coli* and *S. aureus*. Notably, AMP_S4 and AMP_S13 exhibited significant antimicrobial activity against multidrug-resistant ESKAPE pathogens, with MIC values ranging from 2 μg/mL to 16 μg/mL. In comparison, other *de novo* AMP design methods, such as those by Szymczak et al.,^33^ identified only three peptides with comparable activity, while several approaches failed to identify any potent candidates.^12,29,32^ Furthermore, when compared to Pandi et al.,^12^ whose candidate AMPs exhibited limited efficacy against ESKAPE pathogens, AMP_S4 and AMP_S13 demonstrated superior antimicrobial performance, underscoring the effectiveness of our framework.

The bacterial membrane is the primary target for most AMPs,^34^ which exhibit rapid bactericidal activity and a low propensity for resistance development.^35^ Consistent with this mechanism, both AMP_S4 and AMP_S13 rapidly permeabilized bacterial membranes, disrupted the proton motive force (PMF), and increased membrane permeability, leading to bacterial cell death. These findings align with previous studies highlighting the lipid bilayer as a key target for AMP-mediated disruption.^36,37^

A major challenge in the clinical application of linear AMPs is their structural instability, susceptibility to protease degradation, and *in vivo* toxicity.^38^ To address this, we evaluated the stability of AMP_S4 and AMP_S13 under various conditions, including extreme pH, elevated temperatures, protease-rich environments, and plasma exposure. Both peptides retained strong antimicrobial activity under these conditions. Furthermore, cytotoxicity assays demonstrated that AMP_S13 exhibited minimal toxicity toward human cells and low hemolytic activity. *In vivo*, AMP_S13 has been demonstrated to effectively reduce mortality in the *Galleria mellonella* MRSA-infection model and to reduce bacterial load with enhanced wound healing in a murine skin MRSA infection model. Its therapeutic effects were comparable to mupirocin, a widely used antibiotic for treating MRSA-associated skin infections, highlighting its potential as a novel antibacterial agent.

In summary, this study establishes a robust framework for AI-driven generation and screening of AMPs, addressing key challenges in combating antibiotic resistance. BroadAMP-GPT is not limited to the specific strains tested in this study and can be adapted to design and evaluate AMPs targeting diverse pathogens or specific conditions. By thoroughly exploring the design-function landscape of AMPs, BroadAMP-GPT accelerates the discovery of novel peptide-based therapeutics, paving the way for future advancements in antimicrobial drug development.

## Methods

### Data preparation

Five types of datasets were prepared: AMP dataset, non-AMP dataset, MIC dataset, pre-trained AMP dataset, and fine-tuning dataset.

#### AMP dataset

AMP sequences were obtained from five primary public AMP databases: dbAMP,^21^ APD,^22^ CAMP,^23^ SATPdb^24^ and DBAASP,^25^ covering most AMP sequences (downloaded in April 2023). These datasets were merged, and sequences longer than 300 AAs or shorter than 5 AAs were excluded. Duplicates and X-containing sequences were removed, resulting in 28,508 non-redundant sequences.

#### Non-AMP dataset

The dataset was retrieved from UniProt^30^ (http://www.uniprot.org) by setting the ‘subcellular location’ filter to cytoplasm and excluding entries with keywords like antimicrobial, antibiotic, antiviral, antifungal, effector or excreted (downloaded in April 2023). Duplicated sequences were deleted, and only peptides shorter than 300 AAs were kept. Sequences identical to the AMP dataset were also removed. Finally, sequences with a distribution matching the AMP dataset were retained, leaving 28,513 non-AMP sequences.

#### MIC dataset

MIC data for E. coli and S. aureus were collected from dbAMP^21^ (downloaded in April 2023). Sequence and their MIC values (converted to µg/mL) were recorded when the targets column included MIC data. A total of 3,682 sequences were collected for the E. coli MIC dataset, while the S. aureus MIC dataset contained 6,106 sequences. For E. coli, AMPs with MIC values <16 µg/mL (1,368 sequences) were categorized as low-MIC AMPs, and those 16 µg/mL (2,314 sequences) were high-MIC AMPs. For S. aureus, the cutoff was 32 µg/mL, resulting in 3121 low-MIC AMPs and 2985 high-MIC AMPs. These datasets were split into training and test sets at an 8:2 ratio for the MIC prediction model.

#### Pre-trained AMP dataset

The pre-trained AMP dataset was created by excluding duplicate sequences from the large dataset and sequences duplicated from the E. coli MIC dataset. The E. coli MIC dataset had a maximum sequence length of 132 AAs. To maintain length consistency between the pretrained and fine-tuning datasets, sequences shorter than 5 or longer than 132 AAs were removed, leaving 26,545 AMPs.

#### Fine-tuning dataset

The E. coli MIC dataset was utilized for fine-tuning. There were 2,592 overlapping sequences (70% overlap) between the S. aureus and E. coli MIC datasets, meaning the fine-tuned model produces peptides valid for both.

#### Data splitting

The AMP and non-AMP datasets were divided into training-validation and test datasets at a ratio of 8:2. The former was further split into training and validation datasets (8:2). The training dataset included 18,244 AMPs and 18,248 non- AMPs, the test dataset had 5,702 AMPs and 5,703 non-AMPs, and the validation dataset contained 4,562 AMPs and 4,562 non-AMPs for hyperparameter tuning.

#### Aas tokenization

In the AMP prediction model, the peptides (IUPAC single-letter symbols) within the training dataset were treated as tokens. Special class tokens “CLS” and “SEP” were added at the start and end of the sequences, respectively, to demarcate sentence boundaries. Padding “PAD” tokens were used for sequences shorter than 300, standardizing all to 300 tokens. These embeddings were converted into 1024-dimensional vectors for AMP classification.

### Implementation of AMP generation model

AAs of peptides were regarded as molecular units, and AMPs were created using a molecular generation model. The BroadAMP-GPT’s generation model was a mini basically version of the Generative Pre-Training Transformer (GPT) model.^26^ The model employs autoregressive manner to sequentially generate AA tokens in the peptide. Each subsequent token is determined by the previously generated sequence, with masked multihead self-attention applied to prevent information leakage from non-encoded characters in the sequence. It was made up of eight decoder blocks, with each block containing a masked multihead self-attention layer and a fully connected neural network. Each self-attention layer returned a vector of size 256 that was taken as input by the fully connected network. The hidden layer of the neural network outputted a vector of size 1024 and passes it through GELU activation layer. The final layer of the fully connected neural network returned a vector of size 256, that was then used as input for the next decoder block. The masked multihead self-attention layer is the core component of the model, composed of several “Scaled Dot Product Attention”, which enables the model to sequentially capture critical information. Attention can be represented by the following formula:

(1) $$\mathrm{Attention}(Q,K,V)=\mathrm{softmax}\left(\frac{QK^{T}}{\sqrt{d_{k}}}\right)\;$$

where *Q*, *K*, and *V* represent query, key, and value vectors, respectively. *d*_*k*_ is the dimension of *Q* and *K* vectors, and *T* is transpose of the matrix. Since the attention layer processes the entire sequence simultaneously, positional encoding is added into each token to preserve the linear sequence order information.

To indicate the start or end of sequences during sampling, special tokens “CLS” and “SEP” are defined as the start token and the end token, respectively. During the training process, the model was first pre-trained using the pre-trained AMP dataset to learn the fundamental distribution characteristics of AMPs. Subsequently, fine-tuning was performed using the fine-tuning dataset (i.e., further training) to optimize the model’s performance for broad-spectrum AMP generation tasks, thereby enhancing its adaptability and effectiveness in producing broad-spectrum AMPs. At the generation stage, the model receives a starting token and predicts the next token to create a peptide sequence. Ultimately, 100,000 peptide sequences were sampled. During pre-training, the model runs for 30 epochs with a learning rate of 6e-4, while during fine-tuning, it runs for 20 epochs with a learning rate of 1e-5. The AdamW^39^ optimizer was used with beta1 set to 0.9 and beta2 to 0.95.

### Implementation of AMP prediction model

We trained AMP prediction model to distinguish AMPs from non-AMPs. The framework of this model was illustrated in Figure 1(b). NLP algorithms were applied to construct this model. The ProtBERT,^27^ derived from the original BERT^40^ architecture, was pre-trained on approximately 217 million unique protein sequences from the Big Fantastic Database (BFD).^41,42^ This transformer-based model comprises 16 attention heads and 30 hidden layers. As previously mentioned, each AA can be likened to a word and each peptide to a sentence in NLP. Transformer-based models process all components simultaneously, making it difficult to directly measure token relationships. To address this, sinusoidal positional encoding provides positional context. Before training, each word is tokenized, and information about its position in the given sentence is encoded into the initial embedding vector before being processed. During pretraining phase, the masked language modeling (MLM) objective was employed. Here, 15% of the AAs in sequences were masked, challenging the model to predict these hidden elements based on the surrounding AAs. The attention layer (Formula 1) is also a pivotal component of ProtBERT, enabling the modeling of long-range dependencies among sequence elements regardless of their relative positions. By integrating token embeddings, the pre-trained model captures hierarchical information and token relationships through self-attention mechanism, treating each sequence as a “document”.

Our approach was distinct from the fine-tuning process used for per-sequence tasks with the ProtBERT^27^ model, which utilizes global average pooling on the embeddings from BERT’s last hidden state for downstream prediction tasks. This method may lead to a loss of valuable information that was important for making predictions about entire sequences. We retained all outputs as inputs for the subsequent layer. The complete workflow operates as follows: we input the AAs tokenization sequences and positional encoding into ProtBERT^27^ to generate embeddings, producing 300 × 1024-dimensional matrixes. The feature matrices are subsequently processed through a series of neural network layers: a 1D convolutional layer employing 64 filters (kernel size = 16, stride = 1, activation: tanh), where each filter performs unidirectional convolutions; a 1D max-pooling layer (window size = 5, stride = 1) that downsamples activations by extracting maximum values within non-overlapping windows, enhancing positional invariance while reducing overfitting; a 100-unit LSTM layer that captures long-range sequence dependencies; and finally a dense layer with sigmoid activation that generates probability outputs constrained to the (0,1) range for binary classification.

Predicted probabilities greater than or equal to 0.998 were classified as AMP, whereas probabilities less than 0.998 were classified as non-AMP. The model was trained using the AdamW^39^ optimizer with a binary cross-entropy loss function, starting with a learning rate of 0.00001 and a batch size of 32, over 12 epochs. The ReduceLROnPlateau scheduler was employed to reduce the learning rate by a factor of 0.1 if the validation accuracy did not improve for four epochs. Various tuning parameters, such as learning rate and batch size, were considered, and hyperparameters were optimized based on accuracy (Acc). Please refer to Supplementary Table S6 for more information.

### Implementation of MIC prediction model

The MIC prediction models were built using the Linformer^28^ modeling framework (Figure 1(c)). It’s innovative approach to attention mechanism through low-rank projection offers a more efficient and scalable solution for handling long sequences, thereby enhancing its applicability in various NLP tasks and beyond.

To start with, we converted the MIC datasets into a fixed-size vector, and the peptide sequences were broken down into individual AAs for tokenization, with each AA represented by a unique identifier. If the raw sequence did not reach the maximum length of the dataset, the sequence vectors will be padded with zeros. The labels vectors were adding the number 1/0 as the classification label of the sequences, indicating low MIC/high, respectively. Then, the model applied a linear projection to the input sequence to reduce the sequence length from n to a smaller dimension k (where k<<n). And Linformer retained the multi-head attention mechanism but applies it to the projected queries and keys,^28^ formulated as:

(2) $$\mathrm{Attention}(QW_{i}^{Q},E_{i}KW_{i}^{K},F_{i}VW_{i}^{V})=\mathrm{softmax}\left(\frac{QW_{i}^{Q}{\left(E_{i}KW_{i}^{K}\right)}^{T}}{\sqrt{d_{k}}}\right)\cdot F_{i}VW_{i}^{V}\;$$

where *Q*, *K*, and *V* represent query, key, and value vectors, respectively. *d*_*k*_ is the dimension of *Q* and *K* vectors, $W_{i}^{Q},W_{i}^{K},W_{i}^{V}$ are learned matrices. *E*_*i*_, *F*_*i*_, are linear projection matrices.

Multi-head attention allowed the model to capture dependencies from different subspaces. Afterward, it gone through a series of processes such as Value Transformation, Value Linear Projection, Linear Transformation and Residual Connection, Layer Normalization and Feed- Forward Neural Network (FFN). Finally, the output of the FFN was passed through another normalization layer. Finally, the output of the FFN was passed through another normalization layer to obtain the probability values.

In order to apply optimized hyperparameters for model, we first performed a search of hyperparameter that was commonly tuned with DL models: learning rate. The search spaces for each hyperparameter and the corresponding prediction results for an 8:2 train/validation split set of the data were presented in Supplementary Table S7. The search results showed that the hyperparameter setting of learning rate = 1e-3 led to the best prediction performance in terms of accuracy. Therefore, all evaluations and subsequent analyses for MIC model were completed with the above hyperparameters. Additional five-fold cross-validation was applied to check for overfitting. All models converged quickly during training. The two models select the parameters of the best fold of area under the curve (AUC) as the final prediction model and the peptide sequences with prediction scores greater than 0.5 (positive) were considered a candidate AMP.

### Evaluation metrics for the prediction model

We used general quantitative indicators: sensitivity (Sens), specificity (Spec), precision (Prec), accuracy (Acc), Matthews correlation coefficient (MCC) and the area under receiver operating characteristic curve (AUROC) to evaluate our prediction models.

(3) $$\mathrm{ACC}=\frac{\mathrm{TN}+\mathrm{TP}}{\mathrm{TN}+\mathrm{TP}+\mathrm{FN}+\mathrm{FP}}\;$$

(4) $$\mathrm{Sens}=\frac{\mathrm{TP}}{\mathrm{TP}+\mathrm{FN}}\;$$

(5) $$\mathrm{Spec}=\frac{\mathrm{TN}}{\mathrm{TN}+\mathrm{FP}}\;$$

(6) $$\mathrm{Prec}=\frac{\mathrm{TP}}{\mathrm{TP}+\mathrm{FP}}\;$$

(7) $$\mathrm{MCC}=\frac{\left(\mathrm{TP}\times \;\mathrm{TN})-(\mathrm{FP}\times \;\mathrm{FN}\right)}{\sqrt{\left(\mathrm{TP}+\mathrm{FP})(\mathrm{TP}+\mathrm{FN})(\mathrm{TN}+\mathrm{FP})(\mathrm{TN}+\mathrm{FN}\right)}}\;$$

where TP, TN, FP and FN denote the number of true positives, true negatives, false positives and false negatives, respectively.

### Determination of antibacterial activity in vitro

*Bacterial strains and growth conditions*: All Gram-negative and Gram-positive bacterial strains (Supplementary Table S8) were cultured in Luria-Bertani (LB) or trypticase soy broth (TSB) agar plates, respectively, and incubated overnight at 37 °C unless specifically indicated. A single colony of the tested bacterial strains was inoculated into medium broth with shaking at 220 rpm for further experiments.

*Minimal inhibitory concentrations*: Antimicrobial susceptibility testing was performed by broth microdilution method for AMPs against Gram-positive and Gram-negative pathogens above mentioned, following the methods of document CLSI M100-ED34.^43^ The MICs were defined as the lowest concentrations that inhibit visible growth of the tested bacteria. *E. coli* ATCC 25,922 and *S. aureus* ATCC 29,213 were served as quality controls.

*Minimum bactericidal concentrations*: Following the determination of the MICs, the samples were subcultured for 24 h at 37℃. Thereafter, 100 μL of the transparent sample medium was taken and spread on MH agar plates, which were then incubated for 16–20 h at 37℃. The lowest concentration that resulted in a reduction in bacterial growth (i.e., less than five colonies) was recorded as the minimum bactericidal concentrations (MBCs).

*Time-killing kinetics*: For time-kill assay, overnight grown culture of *S. aureus* ATCC 29,213 was diluted with TSB broth to achieve a cellular density of 10^7^ CFU/mL. The AMPs were dissolved and diluted with double-distilled water at final concentrations of 0, 0.5×, 1×, 2×, and 4×MIC. The mixture of bacteria and AMPs was hen subjected to incubation at 37℃ with shaking. Subsequently, 100 μL aliquots of the cultures were taken at the 0, 0.5, 1, 2, 3, and 5-hour time points, diluted in 10- to 1000-fold, and plated on TSA plates for overnight incubation. The bacterial colonies were counted to determine the CFU/mL at each designated time point.

### Drug resistance assay

The development of resistance to the selected AMPs against *S. aureus* ATCC 29,213 was assessed over a 28-day period as previously described.^44^ Briefly, the initial MICs of the tested AMPs were obtained from the aforementioned method. Bacterial cultures with 0.5×MIC of the AMPs were diluted and resuspended for the next round resistance induction assay and MIC measurement. For each passage, the MIC of the compounds was reevaluated, and if it increased, the concentration of the compounds in the culture was increased accordingly. Ciprofloxacin and vancomycin were used as comparison controls.

### Membrane depolarization assays

*S. aureus* ATCC 29,213 and *E. coli* ATCC 25,922 were cultured to mid-logarithmic phase and diluted to a concentration of 1 × 10^6^CFU/mL. The cells were centrifuged, washed with HEPES buffer (20 mm glucose, 5 mm HEPES, pH 7.4), and resuspended with HEPES buffer containing 0.1 M KCl in the same volume. The bacteria suspension was incubated with 0.8 μM diSC3(5) for 30 min to achieve a stable fluorescence. An aliquot of 90 µl of the suspension was placed in 96-well black plate and fluorescence intensity were recorded on a microplate reader for 20 min(excitation wavelength = 622 nm, emission wavelength = 673 nm).Then addition 10 μL of peptide solutions were added and fluorescence intensity were recorded for another 40 min.^45^

### Membrane permeability assays

The fluorescent probe 1-N-phenylnaphthylamine (NPN) and propidium iodide (PI) were used to evaluate the ability of peptides-induced the outer membrane and the inner membrane permeability, respectively. For outer membrane permeability assays, *S. aureus* ATCC 29,213 and *E. coli* ATCC 25,922 were cultured overnight and diluted to 10^8^ CFU/mL (OD_600_ = 0.5). The cells were washed, suspended in HEPES buffer (5 mm HEPES, 20 mm glucose, pH 7.4) and incubated with 10 μM NPN in the dark for 30 min. An aliquot of 90 μL of the probed cells were then placed in 96-well black plate and another 10 μL peptide solutions at different concentrations were added to the plate, and then incubated at 37℃ for 1 h. The NPN fluorescence was recorded at the excitation/emission wavelengths of 350/420 nm, respectively. For inner membrane permeability assays, the suspended cells were incubated with PI (20 μg/mL) in the dark for 30 min, and 90 μL of the probed cells were mixed with 10 μL peptide solutions at different concentrations in 96-well black plate and incubated at 37℃ for 1 h. The PI fluorescence was recorded at the excitation/emission wavelengths of 535/615 nm, respectively.^36^

### Scanning electron microscopy (SEM) and transmission emission microscopy (TEM)

*S. aureu*s ATCC 29,213 cells were grown in TSB overnight and subsequently collected by centrifugation. The cells were suspended in PBS containing peptides at a concentration of 32 μg/mL and incubated at 37℃ for 2 h. The suspension was then subjected to centrifugation and washed thrice with PBS. Thereafter, the cells were suspended in glutaraldehyde at a concentration of 4% and incubated at 4℃ overnight. Finally, the bacteria were dehydrated in a series of different ethanol gradients (10%, 30%, 50%, 70%, 90% and 100%) and dried in a 37℃ oven. After drying and gold spraying, the samples were analyzed by SEM.

For ultrathin sectioning, samples were sequentially treated with glutaraldehyde overnight and osmic acid for 2 h. Thereafter, they were subjected to a series of dehydration steps using a combination of 30%, 50%, 70%, 80%, 90% and 100% ethanol/water solutions, each lasting for a duration of 30 minutes, with the addition of acetone for a period of 20 minutes. Following this, the specimens were infiltrated in a series mixture of acetone and Spurr′s resin (1:1 for 1 h, 1:3 for 3 h) and finally embedded in Spurr′s resin overnight. Ultrathin sections of approximately 70 nm thickness were stained with 2% uranyl acetate and lead citrate (10 min each). The images were captured using a TEM.

### Molecular dynamics (MD) simulations

For MD simulations in an aqueous environment, a generic simulation box was constructed using the CHARMM-GUI service.^46^ The 3D peptide structures were generated withAlphaFold2^47^ and solvated in a 10 Å TIP3P water box. Cl^−^ ions were added to neutralize the system’s charge. System neutralization was achieved through Cl^−^ counterions. MD simulations were executed via Bash commands. The system underwent energy minimization using a 2000-step steepest descent followed by a 3000-step conjugate gradient. After initial energy minimization, unconstrained optimization and MD simulations were performed, which included both heating and equilibration steps. Simulations were conducted for 100 ns at a constant temperature of 300 K and a pressure of 1 atm. Root mean square deviation (RMSD) values were calculated post-simulation.

Furthermore, the MD simulations with bacterial membranes using a generic simulation box was also constructed using CHARMM-GUI. The system included a rectangular lipid bilayer patch composed of 120 1-palmitoyl-2-oleoyl-sn-glycero-3-phosphoethanolamine (POPE) and 60 1-palmitoyl-2-oleoyl-sn-glycero-3-(phospho-rac-(1-glycerol)) (POPG) molecules, embedded in an aqueous solvent containing K^+^ and Cl^−^ ions. Ion number was adjusted to achieve 0.15 mol/L concentration and further modified to neutralize the total system charge. The initial box dimensions across the membrane were set to 11 nm. The CHARMM36 force field^48^ was applied to protein, lipids and ions, while the rigid TIP3P model^49^ was used for water.

During final system assembly, peptide structures, generated via AlphaFold2,^47^ were inserted into the aqueous phase of the simulation box, oriented parallel to the membrane plane, and overlapping water molecules were removed. Simulations were executed using the Gromacs program^50^ with default CHARMM-GUI parameters, employing periodic boundary conditions.^51^ The protocol included potential energy minimization, six rounds of equilibration with stepwise removal of positional restraints for peptides and lipids, followed by production runs at ambient pressure and 310 K. MD simulations were run using the Bash command. The peptides were positioned 3.0 nm from lipid bilayer surface. System optimization was conducted using a 5000-step steepest descent, followed by unconstrained optimization and MD simulations. These simulations included both heating and equilibration steps and were run for 80 ns at a constant temperature of 310 K and a pressure of 1 atm.

### Circular dichroism (CD) spectroscopy

AMP solutions at 100 μg/mL were analyzed in water and SDS (25 mmol/L), at 25℃. Circular dichroism spectra were obtained by averaging three accumulations from 260 to 190 nm, using a quartz cuvette with an optical path length of 1.0 mm, step resolution of 0.5 nm, scan speed of 50 nm/min, and 1s response time. Similar experiments with the respective blank solutions were performed for background subtraction. The content of the secondary structures was estimated with the CDNN software (version 2.1).

### Amps stability analysis

To understand stability of peptide under different pH conditions, AMP solutions were prepared at different pH values (pH = 2, 4, 6, 8, 10, and 12) and then incubated at room temperature for 1 h. For the temperature stability, AMP solutions were incubated at 20, 25, 40, 60, 80, and 100℃ for 1 h. For the protease stability, AMP solutions were incubated with pepsin, trypsin, and proteinase K at different concentration of 10 μg/mL at 37℃ for 1 h.^52,53^ After incubation, the MIC values peptides were determined using the MIC experimental protocol. For plasma stability, the MIC values were determined in the presence of 50% rabbit plasma by a standard MIC assay.^54^

### Cytotoxicity assays

The cytotoxicity of selected AMPs to human laryngeal carcinoma epithelial cell (HEp-2) and colorectal adenocarcinoma cell (Caco-2) lines was evaluated using the CCK-8 method as previously described.^55^ The cells were cultured in high-glucose dulbecco’s modified eagle medium (DMEM) supplemented with 10% fetal bovine serum (FBS) and 1% penicillin-streptomycin. Cells were inoculated in 96-well sterile microtiter plate at 5000 cells/100 μL. After cultured (37℃, 5%CO_2_) for 24 h, cells were treated with AMPs at concentrations ranging from 8 to 128 μg/mL for another 48 h. Afterward, the culture medium was replaced by fresh DMEM medium containing 10% CCK-8 and incubated for 1 h. The absorbance of the solution was measured at 450 nm by a microplate reader. Cell viability was calculated as: % cell viability = [(Abs_sample_ - Abs_blank_)/(Abs_negative_ - Abs_blank_)]×100%. Each experiment was carried out with three replicates.

### Haemolysis assays

Red blood cells (RBCs) from Kunming mice were used to evaluate the hemolytic toxicity of the selected AMPs. Fresh red blood cells were collected from mice and washed until the supernatant was completely cleared. Added 19-fold volume of PBS to prepare 2% RBC suspension. Then 50 μL of RBC suspension was added to 50 μL of peptides at various concentration (1×MIC-16×MIC), followed by incubation at 37℃ or 1 h. Afterward, the mixture was centrifuged at 1500 rpm for 10 min. Then transferred 30 µL supernatant to 96-well plate containing 70 µL PBS. The absorbance of the sample was measured at 450 nm by a microplate reader. PBS and 2% Triton X-100 was used as negative and positive control, respectively. The experiment was performed in triplicate and the hemolysis activity was calculated by the formula hemolysis = [(Absorbance_sample_- Absorbance_PBS_)/(Absorbance_Triton x-100_-Absorbance_PBS_)]×100%.

### Ethics statement

Animal experiments were conducted at the Laboratory Animal Center of Nanjing Agricultural University, according to the guidelines of Experimental Animal Management Measures of Jiangsu Province and were approved by the Laboratory Animal Monitoring Committee of Jiangsu Province, China [Permit number: SYXK (Su) 2021–0086].

### Determination of antibacterial activity in vivo

*Galleria mellonella larvae MRSA-infection model*: *Galleria mellonella* larvae (*n* = 10) were infected with MRSA ATCC 43,300 (1 × 10^4^ CFU/mL, 10 µL) in the right posterior gastropod. The larvae were then injected with AMP_S13 (20 mg/kg, 10 mg/kg, 10 μL) or PBS at 1 h post infection. Larvae were monitored for survival until 72 hours post infection.

*Mouse skin MRSA-infection model*: Mouse topical wound infection model was assessed as described previously.^56^ Female ICR mice (3–4 weeks old, 20 ± 2 g weight) were randomly separated into different groups (*n* = 8) and anesthetized with isoflurane. The backs of the mice were shaved and excised the skin region with fine scissors to create a circular wound with a diameter of 10 mm. A mid-logarithmic growth-phase culture of MRSA YZSA-24 was diluted in MH broth at a density of 10^8^ CFU/mL and 50 µL of the diluted culture was placed on the skin to initiate the infection. After 1, 12, and 24 h post-infection, a volume of 40 μL containing 1% (W/V, 10 mg/mL), 0.5 % (W/V, 5 mg/mL), 0.1 % (W/V, 1 mg/mL) peptides were placed on the wounds. Mupirocin (0.1% W/V, 1 mg/mL) and vehicle control was given at the same time. The images of wounds were captured on day 1, 3, 5, 7, 9, and 11. On day 5 and 11, one mouse from group was euthanized and skin wound was collected for histological H&E staining. On day 11, the mice were euthanized and the size of wounds were measured. Then skin tissue was collected and homogenized with sterile PBS (0.25 g skin tissue in 4 ml PBS). The bacteria load per tissue was calculated by plating diluted skin homogenates on TSB agar plates and counting colonies.

## Supplementary Material

Supplementary Tables and Figures.docx

## Data Availability

The data that support the findings of this study are available in repository name at https://github.com/LYRHeidi/BroadAMP-GPT. These data were derived from the following resources available in the public domain: AMP data were mainly collected from five public AMP databases that include dbAMP^21^ (https://awi.cuhk.edu.cn/dbAMP/.), APD^22^ (http://aps.unmc.edu.), CAMP^23^ (http://www.camp.bicnirrh.res.in.), SATPdbs^24^ (http://crdd.osdd.net/raghava/satpdb.) and DBAASP^25^ (http://dbaasp.org). The non-AMP dataset was downloaded from UniProt^30^ (http://www.uniprot.org).
