# Supplementary material for: BroadAMP-GPT: AI-Driven generation of broad-spectrum antimicrobial peptides for combating multidrug-resistant ESKAPE pathogens
Source: Gut Microbes. 2025 Jun 26;17(1):2523811. doi: 10.1080/19490976.2025.2523811 (PMC12203862; doi:10.1080/19490976.2025.2523811)
Supplement: Supplementary Tables and Figures.docx [file KGMI_A_2523811_SM9096.docx]

Supporting Information for **“BroadAMP-GPT: AI-Driven Generation of Broad-Spectrum Antimicrobial Peptides for Combating Multidrug-Resistant ESKAPE Pathogens”**

Yanru Li^a,b^*, Xianghan Xu^a,c^*, Xiaohui Zhang^d^*, Zhihui Xu^e^*, Jiaqi Zhao^c,f^, Ruiyu Zhu^f^, Ziyu Wang^b^, Wei Ran^c^, Wenqian Zhao^g^, Ningyang Yan^c^, Yifan Leng^c^, Zexu Miao^c^, Xiaomin Wang^c^, Liping Wang^c^, Jinxin Liu^a,f^, Cong Pian^e^, and Jinhu Huang^a,c^

^a^Sanya Institute of Nanjing Agricultural University, Nanjing Agricultural University, Sanya 572025, China; ^b^College of Agriculture, Nanjing Agricultural University, Nanjing 210095 Jiangsu, China; ^c^MOE Joint International Research Laboratory of Animal Health and Food Safety, Risk Assessment Center of Veterinary Drug Residue and Antimicrobial Resistance, Center for Veterinary Drug Research and Evaluation, College of Veterinary Medicine, Nanjing Agricultural University, Nanjing 210095, China; ^d^Department of Veterinary Medicine, School of Tropical Agriculture and Forestry, Hainan University, Haikou 570228, China; ^e^School of Basic Medicine and Clinical Pharmacy, China Pharmaceutical University, Nanjing, 211198, Jiangsu, China; ^f^Laboratory of Gastrointestinal Microbiology, Jiangsu Key Laboratory of Gastrointestinal Nutrition and Animal Health, National Center for International Research on Animal Gut Nutrition, College of Animal Science and Technology, Nanjing Agricultural University, Nanjing 210095 Jiangsu, China; ^g^College of Sciences, Nanjing Agricultural University, Nanjing 210095 Jiangsu, China

CONTACT **Jinhu Huang**, E-mail address: jhuang@njau.edu.cn, address: College of Veterinary Medicine, Nanjing Agricultural University, Nanjing 210095, China;

**Cong Pian**, E-mail address: piancong@njau.edu.cn, address: School of Basic Medicine and Clinical Pharmacy, China Pharmaceutical University, Nanjing, 211198, Jiangsu, China;

**Jinxin Liu**, E-mail address: jxnliu@njau.edu.cn, address: College of Animal Science and Technology, Nanjing Agricultural University, Nanjing 210095, China

*These authors contributed equally to this work.

**Supplementary Tables 1-8**

**Supplementary Figure 1**

**Supplementary Table 1.** The classification performance metrics of the AMP prediction model were evaluated against two other AMP prediction models, utilizing an independent test dataset.

| **Models** | **Sens** | **Spec** | **MCC** | **ACC** | **F1 score** |
| --- | --- | --- | --- | --- | --- |
| AMP classification Model | 0.9907 | 0.9928 | 0.9835 | 0.9918 | 0.992 |
| AMPscanner | 0.8032 | 0.9065 | 0.7136 | 0.8549 | 0.847 |
| LSTM+ATT+BERT | 0.6803 | 1 | 0.7180 | 0.8402 | 0.810 |

Acc: accuracy; Sens: sensitivity; Spec: specificity; MCC: Matthews correlation coefficient.

**Supplementary Table 2**. NCBI BLASTP 2.12.0+ results of 14 candidate AMPs against nonredundant UniProt containing 250 M (250,363,826) sequences. The expect threshold of 10, BLOSUM62 matrix, gap initiation penalty of 11 and gap extension penalty of 1. The AMPs not shown here did not return any hit with an E value threshold of 10^-3^.

| Sequence ID | Score | E-value | %Identity | %Positive |
| --- | --- | --- | --- | --- |
| AMP_S1 | 33.7 | 2.2 | 91 | 91 |
| AMP_ S2 | 32.9 | 9.4 | 63 | 63 |
| AMP_ S3 | 38.8 | 0.073 | 73 | 80 |
| AMP_ S4 | 37.1 | 0.29 | 67 | 67 |
| AMP_ S5 | 36.7 | 0.49 | 100 | 100 |
| AMP_ S6 | 40.9 | 0.005 | 86 | 86 |
| AMP_ S7 | 37.1 | 0.14 | 72 | 72 |
| AMP_ S8 | 36.7 | 0.68 | 79 | 79 |
| AMP_ S9 | 40.1 | 0.050 | 78 | 78 |
| AMP_ S10 | 38.4 | 0.24 | 50 | 50 |
| AMP_ S11 | 36.3 | 1.5 | 73 | 73 |
| AMP_ S12 | 38.8 | 0.22 | 71 | 71 |
| AMP_ S13 | 36.3 | 2.0 | 100 | 100 |
| AMP_ S14 | 34.6 | 8.9 | 92 | 92 |

**Supplementary Table 3**. NCBI BLASTP 2.12.0+ results of 14 candidate AMPs against the training dataset of~0.026 million AMPs. The expect threshold of 10, BLOSUM62 matrix, gap initiation penalty of 9 and gap extension penalty of 1. The AMPs not shown here did not return any hit with an E-value threshold of 10. Note that inferring homology from the E value depends on the dataset size such that for the BLAST against the training dataset the significance threshold would be around the E value of 10^-6^. The AMPs not shown here did not return any hit with an E value threshold of 10^-6^.

| Sequence ID | Score | E-value | %Identity | %Positive |
| --- | --- | --- | --- | --- |
| AMP_S1 | 21.9 | 0.098 | 91 | 100 |
| AMP_S3 | 18.1 | 5.9 | 67 | 75 |
| AMP_ S4 | 17.7 | 4.9 | 58 | 83 |
| AMP_ S5 | 18.9 | 3.0 | 50 | 64 |
| AMP_ S6 | 29.6 | 1e-04 | 79 | 79 |
| AMP_ S7 | 30.0 | 9e-05 | 79 | 79 |
| AMP_ S8 | 21.6 | 0.33 | 63 | 69 |
| AMP_ S9 | 18.9 | 4.8 | 54 | 69 |
| AMP_ S10 | 19.6 | 1.2 | 82 | 91 |
| AMP_ S11 | 18.9 | 2.3 | 41 | 59 |
| AMP_ S12 | 19.6 | 1.7 | 67 | 87 |
| AMP_ S14 | 20.4 | 0.80 | 46 | 85 |

**Supplementary Table 4**. Comparison of MIC and MBC values for AMP_S4 and AMP_S13 in MDR bacterial strains.

| **Bacterial species** | **Strain Description** | **AMP_S4** | | | **AMP_S13** | | |
| --- | --- | --- | --- | --- | --- | --- | --- |
|  |  | **MBC** | **MIC** | **MBC/MIC** | **MBC** | **MIC** | **MBC/MIC** |
| ***S. aureus*** | **ATCC 29213** | **4** | **4** | **1** | **8** | **8** | **1** |
| ***S. aureus*** | **YZSA-15; *ermC, vanB*** | **4** | **4** | **1** | **8** | **8** | **1** |
| ***S. aureus*** | **YZSA-21; *ermC*** | **4** | **4** | **1** | **8** | **8** | **1** |
| ***S. aureus*** | **YZSA-24; *ermC,* *mecA*** | **16** | **16** | **1** | **16** | **16** | **1** |
| ***S. aureus*** | **M92-1; *ermB, tetL, optrA*** | **4** | **4** | **1** | **8** | **8** | **1** |
| ***S. aureus*** | **M147-2; *ermB, tetL, cfr*** | **4** | **4** | **1** | **8** | **8** | **1** |
| ***E. faecium*** | **HFY01** | **4** | **4** | **1** | **4** | **4** | **1** |
| ***E. faecium*** | **QF31; *ermB, cfr*** | **4** | **4** | **1** | **4** | **4** | **1** |
| ***E. faecium*** | **MC38; *ermB,* *optrA*** | **4** | **4** | **1** | **4** | **4** | **1** |
| ***E. faecalis*** | **JH2-2** | **8** | **8** | **1** | **8** | **8** | **1** |
| ***E. faecalis*** | **ES01; *ermB, vanB*** | **8** | **8** | **1** | **8** | **8** | **1** |
| ***E. faecalis*** | **SF-23-1; *ermB, tetL,* *cfr*** | **8** | **8** | **1** | **8** | **8** | **1** |
| ***E. faecalis*** | **J2; *ermB, optrA*** | **8** | **8** | **1** | **8** | **8** | **1** |
| ***S. suis*** | **P1/7** | **4** | **4** | **1** | **4** | **4** | **1** |
| ***S. suis*** | **SH0918; *ermB, tetO, optrA*** | **4** | **4** | **1** | **4** | **4** | **1** |
| ***S. pyogenes*** | **DL02; *ermB*** | **4** | **4** | **1** | **4** | **4** | **1** |
| ***S. agalactiae*** | **DL03; *ermB*** | **4** | **4** | **1** | **4** | **4** | **1** |
| ***E. coil*** | **ATCC 25922** | **8** | **8** | **1** | **8** | **8** | **1** |
| ***S. enteritis*** | **S1; MDR** | **16** | **16** | **1** | **16** | **16** | **1** |
| ***K. pneumoniae*** | **9-1; MDR** | **16** | **16** | **1** | **16** | **16** | **1** |
| ***A. baumannii*** | **AB11** | **2** | **2** | **1** | **2** | **2** | **1** |
| ***A. baumannii*** | **7979; CRAB** | **2** | **2** | **1** | **2** | **2** | **1** |
| ***P. aeruginosa*** | **PAO1** | **8** | **8** | **1** | **8** | **8** | **1** |
| ***P. aeruginosa*** | **PA14** | **8** | **8** | **1** | **8** | **8** | **1** |

**Supplementary Table 5**. Cytotoxicity and hemolytic toxicity of AMP_S4 and AMP_S13.

| Peptides | CC_50_(µg/mL) | | HC_50_ (µg/mL) | CC_50_/MIC^a^ | | HC_50_/MIC^a^ |
| --- | --- | --- | --- | --- | --- | --- |
|  | HEp-2 | Caco-2 |  | HEp-2 | Caco-2 |  |
| AMP_S4 | 35.16 | 15.82 | 18.25 | 8.8 | 3.96 | 4.56 |
| AMP_S13 | 353.3 | 1011 | 928.6 | 44.16 | 126.38 | 116.08 |

MIC values against *S. aureus* ATCC 29213

**Supplementary Table 6**. Prediction performance metrics of the hyperparameter grid search for AMP classification model.

| learning_rate | batch size | val_accuracy |
| --- | --- | --- |
| **1.00E-05** | **32** | **0.9916** |
| 1.00E-05 | 64 | 0.9910 |
| 1.00E-05 | 128 | 0.9887 |
| 1.00E-06 | 32 | 0.9809 |
| 1.00E-06 | 64 | 0.9440 |
| 1.00E-06 | 128 | 0.9431 |

**Supplementary Table 7**. Prediction performance metrics of the hyperparameter grid search for MIC classification model.

|  | **learning_rate** | **val_accuracy** |
| --- | --- | --- |
| *S. aureus* model | **1.00E-03** | **0.69** |
|  | 1.00E-04 | 0.67 |
|  | 1.00E-05 | 0.65 |
| *E. coli* model | **1.00E-03** | **0.74** |
|  | 1.00E-04 | 0.72 |
|  | 1.00E-05 | 0.70 |

**Supplementary Table 8**. Bacterial strains and cells used in this study.

| **Strain** | **Characteristics** | **Reference** |  |
| --- | --- | --- | --- |
| ***S. aureus*** |  |  |  |
| ATCC 29213 | Antibiotic-susceptible type strain |  |  |
| YZSA-15 | *ermC*, *VanB* | This study |  |
| YZSA-21 | *ermC* | This study |  |
| YZSA-24 | mecA, *ermC* | This study |  |
| M92-1 | *ermB*, *tetL*, *optrA*, | This study |  |
| M147-2 | *ermB*, *cfr*, *tetL* | This study |  |
| 13-1 | Clinical isolate | This study |  |
| 14-1 | Clinical isolate | This study |  |
| 14-2 | Clinical isolate | This study |  |
| 15-1 | Clinical isolate | This study |  |
| 37-1 | Clinical isolate | This study |  |
| 50-1 | Clinical isolate | This study |  |
| 51-1 | Clinical isolate | This study |  |
| HS2 | Clinical isolate | This study |  |
| HS3 | Clinical isolate | This study |  |
| HS8 | Clinical isolate | This study |  |
| M2 | Clinical isolate | This study |  |
| M4 | Clinical isolate | This study |  |
| M10 | Clinical isolate | This study |  |
| M12 | Clinical isolate | This study |  |
| M13-2 | Clinical isolate | This study |  |
| M26 | Clinical isolate | This study |  |
| M31 | Clinical isolate | This study |  |
| M33 | Clinical isolate | This study |  |
| M41 | Clinical isolate | This study |  |
| M45 | Clinical isolate | This study |  |
| M46 | Clinical isolate | This study |  |
| M60 | Clinical isolate | This study |  |
| M77 | Clinical isolate | This study |  |
| M80 | Clinical isolate | This study |  |
| M98 | Clinical isolate | This study |  |
| M103 | Clinical isolate | This study |  |
| M106 | Clinical isolate | This study |  |
| M112 | Clinical isolate | This study |  |
| M114 | Clinical isolate | This study |  |
| M117 | Clinical isolate | This study |  |
| M134 | Clinical isolate | This study |  |
| M140 | Clinical isolate | This study |  |
| M144 | Clinical isolate | This study |  |
| M144-2 | Clinical isolate | This study |  |
| M146-1-2 | Clinical isolate | This study |  |
| M150 | Clinical isolate | This study |  |
| M151 | Clinical isolate | This study |  |
| M152 | Clinical isolate | This study |  |
| M153 | Clinical isolate | This study |  |
| M154-2 | Clinical isolate | This study |  |
| ***E. faecium*** |  |  |  |
| HFY01 | Antibiotic-susceptible type strain | This study |  |
| QF31 | *ermB*, *cfr* | This study |  |
| MC38 | *ermB*, *optrA* | This study |  |
| ***E. faecalis*** |  |  |  |
| JH2-2 | Beta-lactams resistance | 1 |  |
| SF23-1 | *ermB*, *tetL*, *cfr* | This study |  |
| J2 | *ermB*, *optrA* | This study |  |
| ES01 | *ermB*, *VanB* | This study |  |
| ***S. suis*** |  |  |  |
| P1/7 | Antibiotic-susceptible type strain | 2 |  |
| SH0918 | *ermB*, *tetO*, *optrA* | 2 |  |
| ***S. pyogenes*** |  |  |  |
| DL02 | *ermB* | This study |  |
| ***S. agalactiae*** |  |  |  |
| DL03 | *ermB* | This study |  |
| ***E. coli*** |  |  |  |
| ATCC 25922 | Antibiotic-susceptible type strain |  |  |
| XM01 | Clinical isolate | This study |  |
| XM02 | Clinical isolate | This study |  |
| XM03 | Clinical isolate | This study |  |
| XM04 | Clinical isolate | This study |  |
| XM05 | Clinical isolate | This study |  |
| XM06 | Clinical isolate | This study |  |
| XM07 | Clinical isolate | This study |  |
| XM08 | Clinical isolate | This study |  |
| XM09 | Clinical isolate | This study |  |
| XM10 | Clinical isolate | This study |  |
| XM11 | Clinical isolate | This study |  |
| BT01 | Clinical isolate | This study |  |
| BT02 | Clinical isolate | This study |  |
| BT03 | Clinical isolate | This study |  |
| BT04 | Clinical isolate | This study |  |
| BT05 | Clinical isolate | This study |  |
| BT06 | Clinical isolate | This study |  |
| BT07 | Clinical isolate | This study |  |
| FMF01 | Clinical isolate | This study |  |
| FMF02 | Clinical isolate | This study |  |
| FMF03 | Clinical isolate | This study |  |
| FMF04 | Clinical isolate | This study |  |
| FMF05 | Clinical isolate | This study |  |
| FMF06 | Clinical isolate | This study |  |
| FMF07 | Clinical isolate | This study |  |
| FMF08 | Clinical isolate | This study |  |
| FMF09 | Clinical isolate | This study |  |
| XXN01 | Clinical isolate | This study |  |
| XXN02 | Clinical isolate | This study |  |
| XXN03 | Clinical isolate | This study |  |
| XXN04 | Clinical isolate | This study |  |
| XXN05 | Clinical isolate | This study |  |
| XXN06 | Clinical isolate | This study |  |
| XXN07 | Clinical isolate | This study |  |
| XXN08 | Clinical isolate | This study |  |
| XXN09 | Clinical isolate | This study |  |
| XXN10 | Clinical isolate | This study |  |
| XXN11 | Clinical isolate | This study |  |
| XXN12 | Clinical isolate | This study |  |
| XXN13 | Clinical isolate | This study |  |
| XXN14 | Clinical isolate | This study |  |
| ***S. enteritis***  S1 | Multidrug-resistant strain | This study |  |
| ***K. pneumoniae*** |  |  |  |
| KP9-1 | Multidrug-resistant strain | This study |  |
| ***A. baumannii*** |  |  |  |
| AB11 | Multidrug-resistant strain | This study |  |
| 7979 | Multidrug-resistant strain | This study |  |
| ***P. aeruginosa***  **PAO1** | Moderate virulent strain | 3 |  |
| **PA14** | Moderate virulent strain | This study |  |
| **Cell lines** | **Characteristics** | **Reference** |  |
| HEp-2 | Human larynx epidermoid carcinoma cells | 2 |  |
| Caco-2 | Human colon epithelial cancer cell | 4 |  |
| **Reference** |  |  |  |
| 1. Wang Y, Lv Y, Cai J, Schwarz S, Cui L, Hu Z, Zhang R, Li J, Zhao Q, He T, et al. A novel gene, optrA, that confers transferable resistance to oxazolidinones and phenicols and its presence in Enterococcus faecalis and Enterococcus faecium of human and animal origin. J Antimicrob Chemother. 2015; 70:2182-90. doi:10.1093/jac/dkv116.  2. Huang J, Dai X, Wu Z, Hu X, Sun J, Tang Y, Zhang W, Han P, Zhao J, Liu G, et al. Conjugative transfer of streptococcal prophages harboring antibiotic resistance and virulence genes. ISME J. 2023; 17:1467-81. doi:10.1038/s41396-023-01463-4.  3. Grace A, Sahu R, Owen DR, Dennis VA. Pseudomonas aeruginosa reference strains PAO1 and PA14: A genomic, phenotypic, and therapeutic review. Front Microbiol. 2022; 13:1023523. doi:10.3389/fmicb.2022.1023523.  4. Song Y, Guan R, Lyu F, Kang T, Wu Y, Chen X. In vitro cytotoxicity of silver nanoparticles and zinc oxide nanoparticles to human epithelial colorectal adenocarcinoma (Caco-2) cells. Mutat Res. 2014; 769:113-8. doi:10.1016/j.mrfmmm.2014.08.001. | | | |


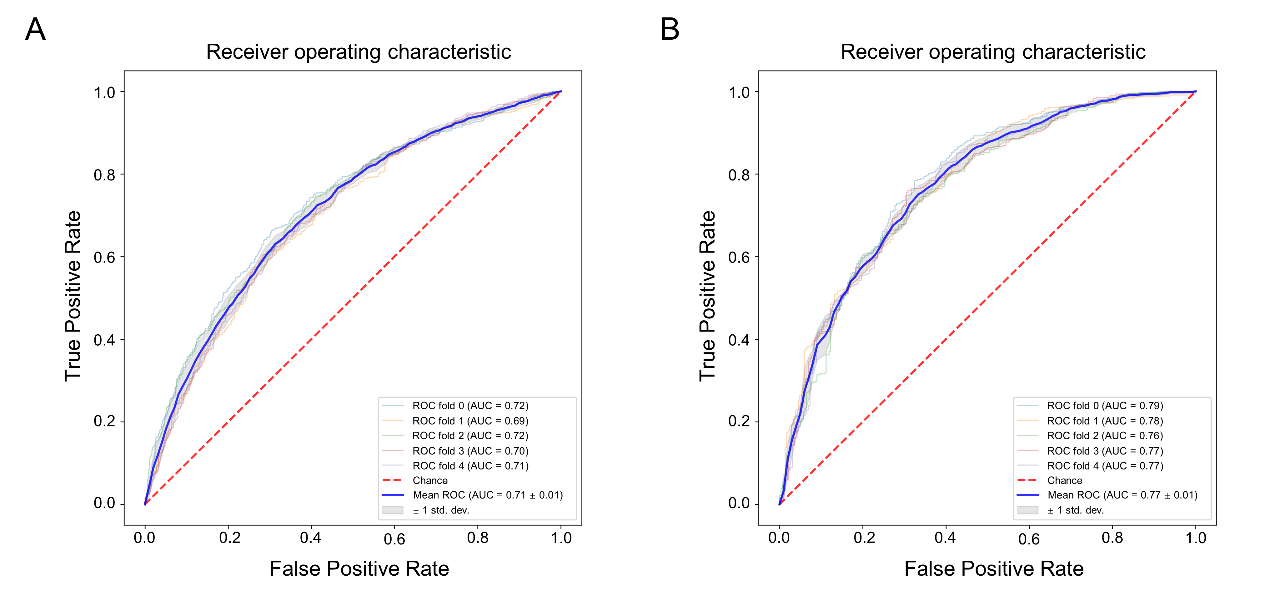


**Supplementary Figure 1**. ROC curves are shown for the five-fold MIC classification models. (A) the *E. coli* MIC classification model, (B) the *S. aureus* MIC classification model.
